# Supplementary material for: Enzymes in the Cholesterol Synthesis Pathway: Interactomics in the Cancer Context
Source: Biomedicines. 2021 Jul 26;9(8):895. doi: 10.3390/biomedicines9080895 (PMC8389681; doi:10.3390/biomedicines9080895)
Supplement: Supplementary file 1 [file biomedicines-09-00895-s001.zip › biomedicines-1303461-Supplementary file #4_Ershov et al-done.pdf]

biomedicines

MDPI

*Supplementary file #4*

# Enzymes in the Cholesterol Synthesis Pathway: Interactomics in the Cancer Context

**Pavel Ershov, Leonid Kaluzhskiy, Yuri Mezentsev, Evgeniy Yablokov, Oksana Gnedenko and Alexis Ivanov**

## Tables

**Table S3.** Tumor-specific prognostic significance of a panel of seventeen cholesterol synthesis enzymes.

| Tumor | Parameter | LogRankP | Hazard Ratio*** | pHR | Cases |
|---|---|---|---|---|---|
| ACC | OS* | 0.16 | 1.80 | 0.16 | 38 |
| | RFS** | 0.021 | 2.20 | 0.024 | 38 |
| BLCA | OS | 0.00079 | 1.70 | 0.00091 | 201 |
| | RFS | 0.022 | 1.50 | 0.023 | 201 |
| BRCA | OS | 0.084 | 1.30 | 0.085 | 535 |
| | RFS | 0.86 | 1.00 | 0.86 | 535 |
| CESC | OS | 0.083 | 1.50 | 0.086 | 146 |
| | RFS | 0.085 | 1.70 | 0.089 | 146 |
| CHOL | OS | 0.54 | 0.74 | 0.54 | 18 |
| | RFS | 0.097 | 0.45 | 0.11 | 18 |
| COAD | OS | 0.60 | 0.88 | 0.6 | 135 |
| | RFS | 0.61 | 0.88 | 0.61 | 135 |
| DLBC | OS | 0.98 | 1.00 | 0.98 | 23 |
| | RFS | 0.32 | 1.90 | 0.32 | 23 |
| ESCA | OS | 0.067 | 0.65 | 0.067 | 91 |
| | RFS | 0.75 | 0.92 | 0.75 | 91 |
| GBM | OS | 0.87 | 1.00 | 0.85 | 81 |
| | RFS | 0.87 | 1.00 | 0.89 | 81 |
| HNSC | OS | 0.15 | 1.20 | 0.16 | 259 |
| | RFS | 0.73 | 0.94 | 0.72 | 259 |
| KICH | OS | 0.098 | 3.50 | 0.12 | 32 |
| | RFS | 0.16 | 2.50 | 0.18 | 32 |
| **KIRC** | OS | **$1.7*10^{-5}$** | **0.51** | **$2.4*10^{-5}$** | **258** |
| | RFS | **0.00014** | **0.49** | **0.00019** | **258** |
| KIRP | OS | 0.92 | 0.97 | 0.92 | 141 |
| | RFS | 0.44 | 1.20 | 0.44 | 141 |
| LAML | OS | 0.077 | 1.60 | 0.08 | 53 |
| | RFS | 1.00 | 1.00 | no | 53 |
| LGG | OS | 0.08 | 0.73 | 0.082 | 257 |
| | RFS | 0.11 | 0.77 | 0.11 | 257 |
| LICH | OS | 0.42 | 1.20 | 0.42 | 182 |
| | RFS | 0.31 | 1.20 | 0.31 | 182 |
| LUAD | OS | 0.66 | 1.10 | 0.66 | 239 |
| | RFS | 0.98 | 1.00 | 0.99 | 239 |

| | | | | | |
|---|---|---|---|---|---|
| LUSC | OS | 0.43 | 1.10 | 0.43 | 241 |
| | RFS | 0.24 | 1.20 | 0.25 | 241 |
| MESO | OS | 0.01 | 1.90 | 0.011 | 41 |
| | RFS | 0.13 | 1.60 | 0.13 | 41 |
| OV | OS | 0.23 | 1.20 | 0.23 | 212 |
| | RFS | 0.59 | 0.93 | 0.59 | 212 |
| PAAD | OS | 0.33 | 1.20 | 0.33 | 89 |
| | RFS | 0.56 | 1.10 | 0.55 | 89 |
| PCPG | OS | 0.29 | 2.50 | 0.3 | 91 |
| | RFS | 0.85 | 1.10 | 0.85 | 91 |
| PRAD | OS | 0.81 | 1.20 | 0.81 | 246 |
| | RFS | 0.22 | 0.77 | 0.22 | 246 |
| READ | OS | 0.46 | 0.70 | 0.46 | 46 |
| | RFS | 0.38 | 0.67 | 0.39 | 46 |
| SARC | OS | 0.002 | 1.90 | 0.0024 | 131 |
| | RFS | 0.021 | 1.50 | 0.02 | 131 |
| SKCM | OS | 0.55 | 1.10 | 0.55 | 229 |
| | RFS | 0.92 | 0.99 | 0.92 | 229 |
| STAD | OS | 0.56 | 1.10 | 0.55 | 192 |
| | RFS | 0.84 | 0.96 | 0.84 | 192 |
| TGCT | OS | 0.30 | 3.10 | 0.33 | 68 |
| | RFS | 0.44 | 1.30 | 0.44 | 68 |
| THCA | OS | 0.36 | 1.60 | 0.36 | 255 |
| | RFS | 0.25 | 0.71 | 0.25 | 255 |
| THYM | OS | 0.11 | 0.29 | 0.14 | 59 |
| | RFS | 0.95 | 0.97 | 0.95 | 59 |
| UCEC | OS | 0.17 | 0.60 | 0.17 | 86 |
| | RFS | 0.14 | 0.60 | 0.14 | 86 |
| UCS | OS | 0.61 | 0.84 | 0.61 | 28 |
| | RFS | 0.14 | 0.58 | 0.14 | 28 |
| UVM | OS | 0.049 | 2.40 | 0.057 | 39 |
| | RFS | 0.0075 | 3.70 | 0.012 | 39 |

*Overall survival (OS); **Disease-free survival (RFS); ***Hazard ratio in a high expression group

**Table S4.** Pan-cancer prognostic significance of each cholesterol synthesis enzymes.

| Gene name | Parameter | LogRankP | Hazard Ratio*** | pHR | Cases |
|---|---|---|---|---|---|
| HMGCR | OS* | 0.26 | 1.00 | 0.26 | 4750 |
| | RFS** | 0.021 | 0.92 | 0.021 | 4750 |
| MVK | OS | $2.0*10^{-13}$ | 0.76 | $2.4*10^{-13}$ | 4750 |
| | RFS | $7.9*10^{-14}$ | 0.75 | $9.9*10^{-14}$ | 4750 |
| PMVK | OS | 0 | 0.72 | 0 | 4750 |
| | RFS | $1.1*10^{-5}$ | 0.85 | $1.1*10^{-5}$ | 4750 |
| MVD | OS | $2.1*10^{-6}$ | 1.20 | $2.2*10^{-6}$ | 4750 |
| | RFS | $1.7*10^{-8}$ | 1.20 | $1.8*10^{-8}$ | 4750 |
| FDPS | OS | $5.1*10^{-14}$ | 1.30 | $6.2*10^{-14}$ | 4751 |

| | | | | | |
|---|---|---|---|---|---|
| | RFS | $7.8*10^{-16}$ | 1.40 | $1.0*10^{-15}$ | 4751 |
| FDFT1 | OS | 0.00028 | 1.10 | 0.00028 | 4750 |
| | RFS | 0.021 | 1.10 | 0.021 | 4750 |
| SQLE | OS | 0 | 1.40 | 0 | 4751 |
| | RFS | $1.3*10^{-8}$ | 1.20 | $1.3*10^{-8}$ | 4751 |
| LSS | OS | $3.4*10^{-6}$ | 1.20 | $3.5*10^{-6}$ | 4750 |
| | RFS | $2.7*10^{-7}$ | 1.20 | $2.9*10^{-7}$ | 4750 |
| DHCR24 | OS | 0.00028 | 1.10 | 0.00029 | 4751 |
| | RFS | 0.47 | 1.00 | 0.47 | 4751 |
| CYP51A1 | OS | 0.00018 | 1.10 | 0.00019 | 4751 |
| | RFS | 0.0011 | 1.10 | 0.0011 | 4751 |
| TM7SF2 | OS | $1.4*10^{-11}$ | 0.78 | $1.5*10^{-11}$ | 4750 |
| | RFS | 0.12 | 0.94 | 0.12 | 4750 |
| MSMO1 | OS | $3.4*10^{-10}$ | 1.30 | $3.7*10^{-10}$ | 4750 |
| | RFS | 0.0081 | 1.10 | 0.0081 | 4750 |
| NSDHL | OS | $5.9*10^{-10}$ | 1.30 | $6.4*10^{-10}$ | 4747 |
| | RFS | 0.0069 | 1.10 | 0.0068 | 4747 |
| HSD17B7 | OS | 0.78 | 0.99 | 0.78 | 4749 |
| | RFS | 0.0069 | 0.90 | 0.0068 | 4749 |
| EBP | OS | 0.0043 | 1.10 | 0.0043 | 4750 |
| | RFS | 0.011 | 1.10 | 0.011 | 4750 |
| SC5D | OS | 0.97 | 1.0 | 0.96 | 4751 |
| | RFS | 0.22 | 1.00 | 0.22 | 4751 |
| DHCR7 | OS | $8.9*10^{-16}$ | 1.30 | $1.1*10^{-15}$ | 4750 |
| | RFS | $8.1*10^{-6}$ | 1.20 | $8.3*10^{-6}$ | 4750 |

*Overall survival (OS); **Disease-free survival (RFS); ***Hazard ratio in a high expression group

**Figures**

A

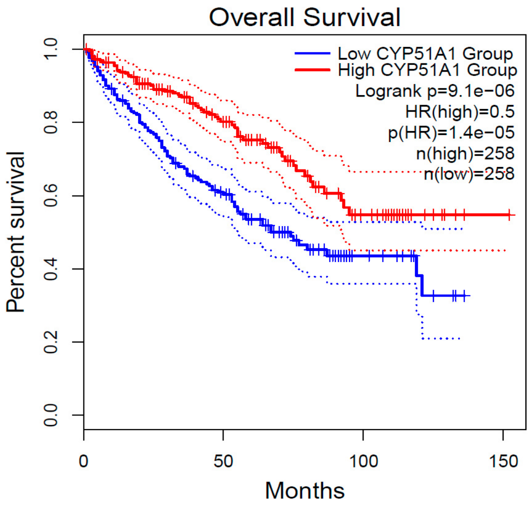


B

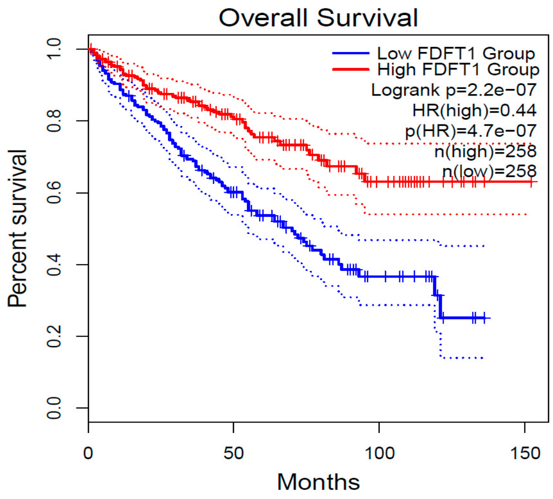


C

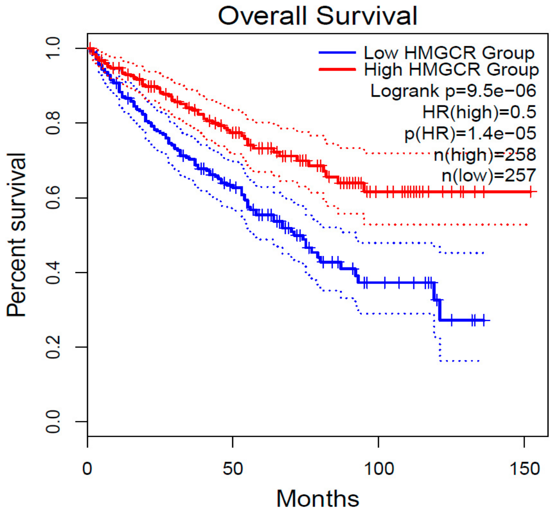


D

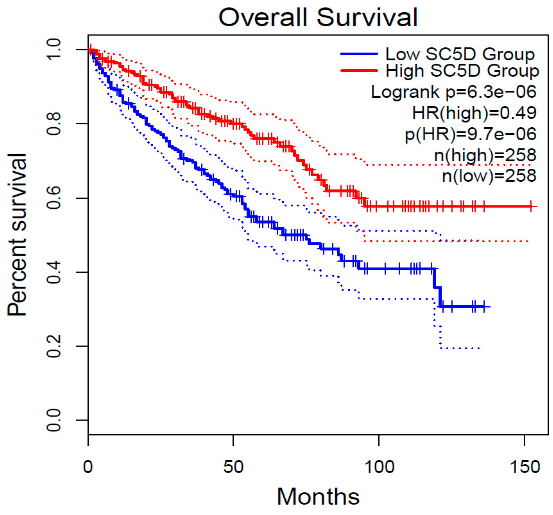


E

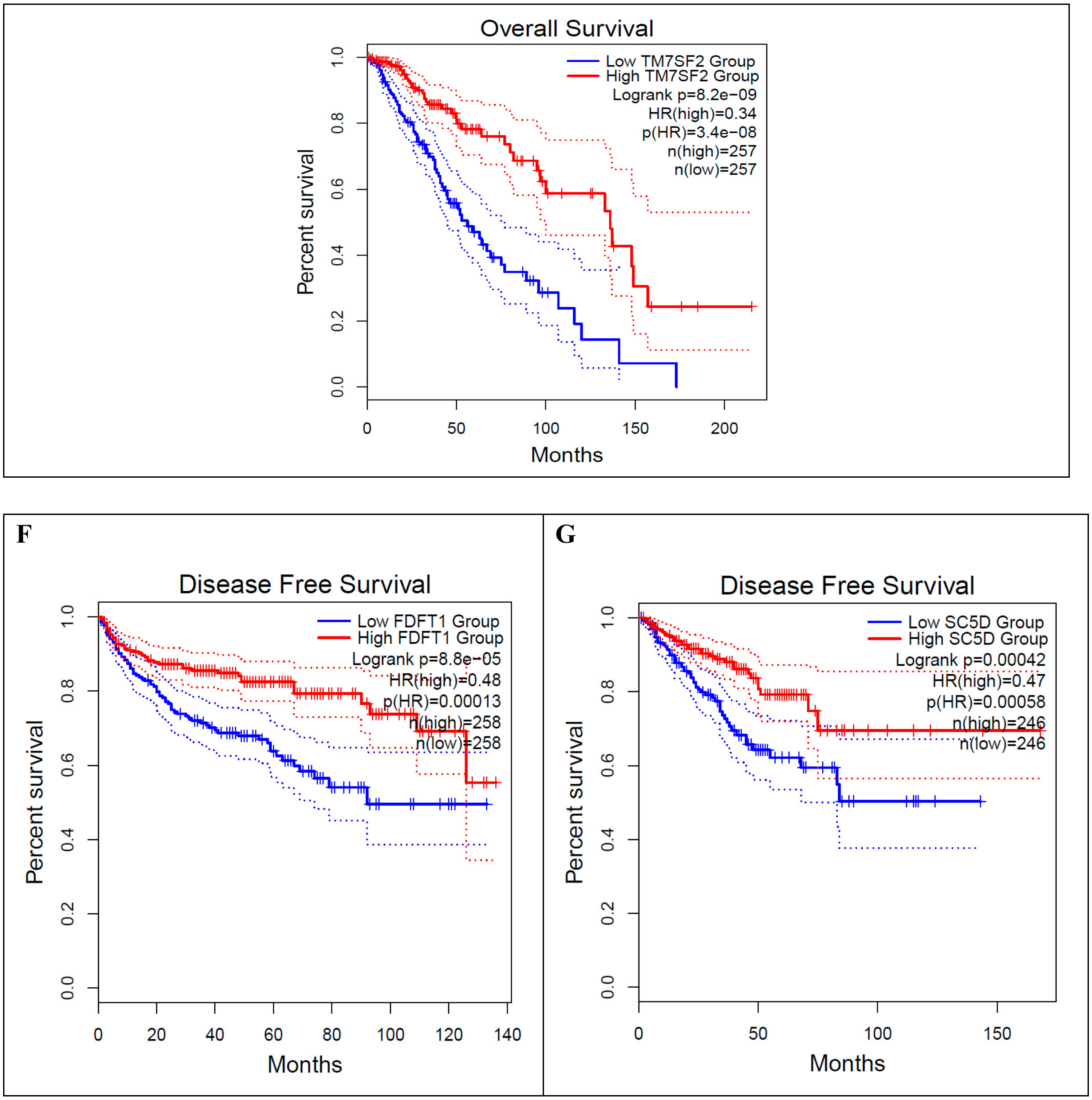


**Figure S9.** The Kaplan-Meier plots for genes of cholesterol biosynthesis pathway meeting the selection criteria: p<0.001, Hazard ratio (HR) ≤ 0.5 or HR ≥ 2 as well as a number of cases ≥ 200. Overall survival: CYP51A1 in KIRC (**A**), FDFT1 in KIRC (**B**), HMGCR in KIRC (**C**), SC5D in KIRC (**D**), TM7SF2 in LGG (**E**); disease free survival: FDFT1 in KIRC (**F**), SC5D in PRAD (**G**)

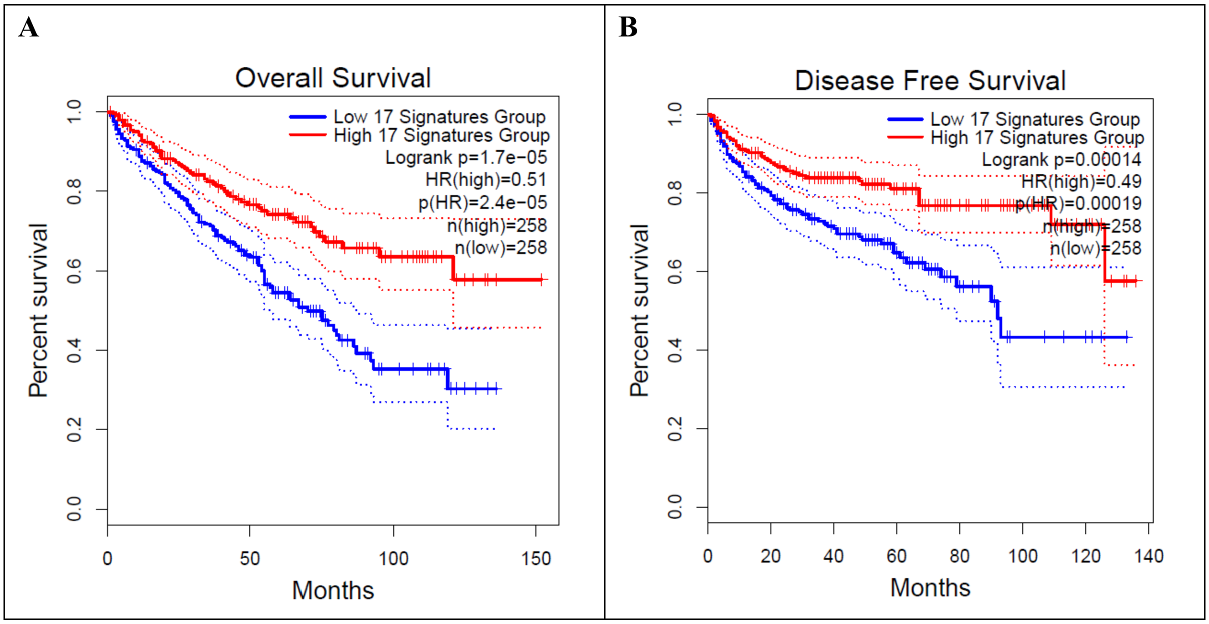


**Figure S10.** The Kaplan-Meier plots for panel of seventeen cholesterol synthesis enzymes in KIRC, overall (**A**) and disease free (**B**) survival.
