# Supplementary material for: Enzymes in the Cholesterol Synthesis Pathway: Interactomics in the Cancer Context
Source: Biomedicines. 2021 Jul 26;9(8):895. doi: 10.3390/biomedicines9080895 (PMC8389681; doi:10.3390/biomedicines9080895)
Supplement: Supplementary file 1 [file biomedicines-09-00895-s001.zip › biomedicines-1303461-Supplementary file #1_Ershov et al-done.pdf]

biomedicines

Supplementary file #1

Enzymes in the Cholesterol Synthesis Pathway: Interactomics in the Cancer Context

Pavel Ershov, Leonid Kaluzhskiy, Yuri Mezentsev, Evgeniy Yablokov, Oksana Gnedenko and Alexis Ivanov

Table S1. The main characteristics of the cholesterol synthesis enzymes.

Gene name / Definition	EC number*/ Gene ID/ UniProt ID	Mw, kDa	Subcellular localization**	Enzyme Reaction Product
HMGCR / 3-hydroxy-3-methylglutaryl-CoA reductase	EC:2.7.1.36/3156/P04035	97.5	peroxisomal membrane, ERM***	mevalonate
MVK / mevalonate kinase	EC:2.7.1.36/4598/Q03426	42.5	cytosol, peroxisome	mevalonate-5-phosphate
PMVK / phosphomevalonate kinase	EC:2.7.4.2/10654/Q15126	22.0	cytosol, peroxisome, ERM	mevalonate-5-pyrophosphate (PP)
MVD / mevalonate diphosphate decarboxylase	EC:4.1.1.33/4597/P53602	43.4	cytosol, extracellular	isopentenyl-PP
FDPS / (2E,6E)-farnesyl diphosphate synthase	EC:4.1.1.33/2224/P14324	48.3	cytosol, nucleoplasm	(E,E)-farnesyl-PP
FDFT1 / farnesyl-diphosphate farnesyltransferase 1	EC:2.5.1.21/2222/P37268	48.1	ER, ERM	presqualene-PP, squalene
SQLE / squalene epoxidase	EC:1.14.14.17/6713/Q14534	63.9	ER, ERM, microsoms, cytoplasmic vesicle, cytosol	(S)-squalen-2,3-epoxide
LSS / lanosterol synthase	EC:5.4.99.7/4047/P48449	83.3	cytosol, ERM, lipid droplet	lanosterol
DHCR24 / 24-dehydrocholesterol reductase	EC:1.1.1.270, EC:1.3.1.72/1718/Q15392	60.1	ERM, Golgi membrane, nucleus, cytosole	lanosterol, zymosterole, cholesta-8 en-3-beta-ol
CYP51A1 / sterol 14-alpha-demethylase	EC:1.14.14.154/1595/Q16850	56.8	ERM, microsoms	4,4-dimethyl-cholesta-8,14,24-trienol
TM7SF2 / delta(14)-sterol reductase	EC:1.3.1.70/73166/O76062	46.4	ERM, cytoplasmic vesicle, nuclear inner membrane	4,4-dimethyl-5α-cholesta-8,14,24-trien-3β-ol
MSMO1 / methylsterol monooxygenase 1	EC:1.14.18.9/6307/Q15800	35.2	ER membrane, plasma membrane	4-methylzymosterol-carboxylate, 4-alpha-carboxy-5-alpha-cholesta-8,24-dien-3-beta-ol
NSDHL / sterol-4alpha-carboxylate 3-dehydrogenase	EC:1.1.1.170/50814/Q15738	41.9	ER membrane, lipid droplet	3-keto-4-methylzymosterol, zymosterone
HSD17B7 / 17-beta-estradiol 17-dehydrogenase	EC:1.1.1.270/51478/P56937	38.2	ERM, plasma membrane	4-methylzymosterol
EBP / cholestenol delta-isomerase	EC:5.3.3.5/10682/Q15125	26.3	ERM, nucleus envelope, cytoplasmic vesicles	cholesta-7,24-dien-3-beta-ol, lathosterol

SC5D / sterol-C5-desaturase	EC:1.14.19.20/6309 /O75845	35.3	cytosol, ERM, nucleoplasm	7-dehydrocholesterol
DHCR7 / 7-dehydrocholesterol reductase	EC:1.3.1.21/1717/ Q9UBM7	54.5	cytosol, ERM, nuclear outer membrane	cholesterol

*Enzyme Commission number (EC number). **NextProt database (https://www.nextprot.org/ accessed on 29 March 2021) ***ERM - Endoplasmic Reticulum Membrane

Table S2. Pathways enrichment of protein partners of the cholesterol synthesis enzymes.

GeneSet	Description	Protein Partners
	**KEGG Pathways**	
hsa05230	Central carbon metabolism in cancer	ERBB2, FGFR1, G6PD, GLS, HRAS, IDH1, SLC1A5, TP53
hsa01230	Biosynthesis of amino acids	ALDOC, ASS1, CTH, ENO1, IDH1, MAT1A, SHMT2, TALDO1
hsa04141	Protein processing in endoplasmic reticulum	AMFR, CAPN1, FBXO6, HSP90B1, HSP90B1, HSPA8, MARCH6, PLAA, SEC31A, SYVN1, VCP
hsa05215	Prostate cancer	CREB3, ERBB2, FGFR1, HRAS, HSP90B1, IKBKB, TP53
hsa00900	Terpenoid backbone biosynthesis	PDSS1, PDSS2, ZMPSTE24
hsa04210	Apoptosis	CAPN1, CASP7, HRAS, IKBKB, LMNA, TNFRSF10A, TP53
hsa04725	Cholinergic synapse	CHRM4, CHRM5, CREB3, GNB5, HRAS, PRKACA
hsa04726	Serotonergic synapse	ALOX5, GNB5, HRAS, HTR3C, PRKACA, SLC18A1
hsa01200	Carbon metabolism	ALDOC, ENO1, G6PD, IDH1, SHMT2, TALDO1
	**Panther pathways**	
P02739	De novo pyrimidine deoxyribonucleotide biosynthesis	DTYMK, NME1, NME2
P02738	De novo purine biosynthesis	ADSS, NME1, NME2
P02740	De novo pyrimidine ribonucleotides biosythesis	NME1, NME2
P00003	Alzheimer disease-amyloid secretase pathway	CHRM4, CHRM5, MAPK6, NCSTN
P04375	5HT3 type receptor mediated signaling pathway	HTR3C, SLC18A1
	**Reactome pathways**	
R-HSA-532668	N-glycan trimming in the ER and Calnexin/Calreticulin cycle	AMFR, MARCH6, RNF139, SYVN1, UBC, VCP
R-HSA-901032	ER Quality Control Compartment (ERQC)	AMFR, MARCH6, RNF139, SYVN1, UBC
R-HSA-901042	Calnexin/calreticulin cycle	AMFR, MARCH6, RNF139, SYVN1, UBC
R-HSA-8853336	Signaling by plasma membrane FGFR1 fusions	ERLIN2, FGFR1
R-HSA-6806664	Metabolism of vitamin K	UBIAD1, VKORC1
R-HSA-5683057	MAPK family signaling cascades	DUSP6, ERBB2, FGFR1, FN1, HRAS, KSR1, MAPK6, MOV10, PRKACA, PSME4, RASGRF2, UBC
R-HSA-499943	Interconversion of nucleotide di- and triphosphates	DTYMK, GSR, NME1, NME2
R-HSA-2559585	Oncogene Induced Senescence	ETS1, MOV10, TP53, UBC
R-HSA-112315	Transmission across Chemical Synapses	CHRND, COMT, GLS, GNB5, HRAS, HSPA8, HTR3C, PRKACA, RASGRF2, SLC38A1
R-HSA-449147	Signaling by Interleukins	ALOX5, CA1, DUSP6, FN1, HSP90B1, HSPA8, HSPA9, IKBKB, PITPNA, PRKACA, PSME4, PTPN5, TALDO1, TP53, UBC
	**Wiki pathways**	

WP4290	Metabolic reprogramming in colon cancer	ENO1, G6PD, GLS, SHMT2, SLC1A5, TALDO1
WP3303	RAC1/PAK1/p38/MMP2 Pathway	CASP7, EIF4EBP1, ERBB2, FN1, HRAS, IKBKB, TP53
WP107	Translation Factors	EEF1G, EIF3A, EIF3F, EIF4EBP1, EIF5A, PABPC1
WP100	Glutathione metabolism	G6PD, GSR, GSS, IDH1
WP1982	Sterol Regulatory Element-Binding Proteins (SREBP) signalling	AMFR, INSIG1, INSIG2, PRKACA, RNF139, SEC31A
WP4299	Lamin A-processing pathway	LMNA, ZMPSTE24
WP2525	Trans-sulfuration and one carbon metabolism	CTH, GSS, MAT1A, SHMT2
WP4504	Cysteine and methionine catabolism	CTH, GSS, MAT1A
WP3925	Amino Acid metabolism	ASS1, CTH, GLS, GSR, GSS, IDH1
	**Wiki cancer pathways**	
WP4290	Metabolic reprogramming in colon cancer	ENO1, G6PD, GLS, SHMT2, SLC1A5, TALDO1
WP4155	Endometrial cancer	ERBB2, FGFR1, HRAS, ILK, TP53
WP704	Methylation Pathways	COMT, MAT1A
WP4223	Ras Signaling	ETS1, FGFR1, GNB5, HRAS, IKBKD, KSR1, PRKACA, RASGRF2
WP2828	Bladder Cancer	ERBB2, FGFR, TP53

Table S3. The spectrum of protein partners, which can modify the cholesterol synthesis enzymes.

Modifying protein /PTM	Cancer Hallmarks[1]	Major Signaling Pathways in Cancer[2]	Ref.
	**HMGCR**		
5'-AMP-activated protein kinase (AMPK) /*	Reprogramming energy metabolism, resisting cell death, sustaining proliferative signaling	mTOR, PI3K-Akt, insulin resistance	[1–3]
Ser/Thr protein phosphatase 2A(PP2A) /**	Activating invasion and metastasis, evading growth suppressors, reprogramming energy metabolism, resisting cell death, sustaining proliferative signaling	TGF-beta, PI3K-Akt	[4,5]
Cathepsin L (CTSL) /#	Activating invasion and metastasis, inducing angiogenesis, resisting cell death, sustaining proliferative signaling, tumor-promoting inflammation	Apoptosis, proteoglycans in cancer	[6]
RING finger protein 145 (RNF145) /✦	n/d	n/d	[7]
E3 ubiquitin-protein ligase AMFR (gp78) /✦	n/d	n/d	[8]
Ubiquitin carboxyl-terminal hydrolase 20 (USP20) /✦✦	n/d	n/d	[9]
Galactosyl-xylosyl-protein-3-beta-glucuronosyltransferase 3 (B3GAT3) /■	n/d	n/d	PPI[3]

cAMP-dependent protein kinase (PRKACA) /*	Activating invasion and metastasis, enabling replicative Immortality, evading growth suppressors, evading immune destruction, inducing angiogenesis, reprogramming energy metabolism, sustaining proliferative signaling, tumor-promoting inflammation	Ras, chemokine, Wnt, insulin resistance, proteoglycans in cancer, MAPK	PPI
E3 ubiquitin-protein ligase RNF139 (RNF139) /✦	n/d	n/d	PPI
**MVK**			
Kelch-like protein 10 (KLHL10) /✦	n/d	n/d	PPI
Ser/Thr-protein phosphatase PP1 (PPP1CB) /**	Activating invasion and metastasis, evading growth suppressors, inducing angiogenesis, sustaining proliferative signaling	Insulin resistance, proteoglycans in cancer	PPI
PMVK			
Mannose-P-dolichol utilization defect 1 protein (MPDU1) /■	n/d	n/d	PPI
**MVD**			
Cytosolic non-specific dipeptidase (CNDP2) /#	n/d	n/d	PPI
Cathepsin A (CTSA) /#	Resisting cell death	n/d	PPI
Caspase 7 (CASP7) /#	Resisting cell death, tumor-promoting inflammation	Apoptosis	PPI
Calpain-1 catalytic subunit (CAPN1) /#	Resisting cell death	Apoptosis	PPI
**FDPS**			
Protein phosphatase methylesterase 1 (PPME1) /**	n/d	n/d	PPI
STAM-binding protein (STAMBP) /#	n/d	n/d	PPI
Ubiquitin thioesterase OTUB1 (OTUB1) /✦	n/d	n/d	PPI
Mitogen-activated protein kinase MEK5/ERK5 /*	Activating invasion and metastasis, evading growth suppressors, sustaining proliferative signaling	MAPK	[10]
**FDFT1**			
Protein O-mannosyl-transferase 2 (POMT2) /■	n/d	n/d	PPI
Ubiquitin carboxyl-terminal hydrolase 32 (USP32) /✦✦	n/d	n/d	PPI
E3 ubiquitin-protein ligase HERC2 (HERC2) /✦	n/d	n/d	PPI
Dual specificity protein phosphatase 6 (DUSP6) /**	Activating invasion and metastasis, sustaining proliferative signaling	MAPK, Transcriptional misregulation in cancer	PPI
Ubiquitin carboxyl-terminal hydrolase isozyme L5 (UCHL5) /✦✦	n/d	n/d	PPI

Mitogen-activated protein kinase 6 (MAPK6) /*	n/d	MAPK	PPI
**SQLE**			
Glycogen synthase kinase-3 beta (GSK3Â) /*	Activating invasion and metastasis, enabling replicative Immortality, evading growth suppressors, evading immune destruction, resisting cell death, sustaining proliferative signaling, tumor-promoting inflammation	Chemokine, PI3K-Akt, Wnt, insulin resisitance	[11]
E3 ubiquitin-protein ligase MARCH6 (MARCH6 or Teb4) /✦	n/d	n/d	[12–15]
E3 ubiquitin-protein ligase MYLIP (IDOL) /✦	n/d	n/d	[12]
**CYP51A1**			
E3 ubiquitin-protein ligase MARCH6 (MARCH6) /✦	n/d	n/d	[16]
**MSMO1**			
Transmembrane protease serine 11B (TMPRSS11B) /#	n/d	n/d	PPI
**NSDHL**			
Ubiquitin carboxyl-terminal hydrolase 47 (USP47) /✦✦	n/d	n/d	PPI
**HSD17B7**			
Ser/Thr-protein kinase BRSK1(BRSK1) /*	n/d	n/d	PPI
Tartrate-resistant acid phosphatase type 5 (ACP5) /**	Resisting cell death	n/d	PPI
**DHCR24**			
E3 ubiquitin-protein ligase MARCH6 (MARCH6) /✦	n/d	n/d	[16]
Tyrosine-protein phosphatase non-receptor type 5 (PTPN5) /**	Activating invasion and metastasis, sustaining proliferative signaling	n/d	PPI
Protein kinase C (PKC) /*	Activating invasion and metastasis, enabling replicative Immortality, evading growth suppressors, evading immune destruction, inducing angiogenesis, reprogramming energy metabolism, sustaining proliferative signaling, tumor-promoting inflammation	Ras, HIF-1, mTOR, PI3K-Akt, Wnt, VEGF, MAPK	[17]
E3 ubiquitin-protein ligase (AMFR) /✦	n/d	n/d	[18]
E3 ubiquitin-protein ligase Itchy (Itch) /✦	Resisting cell death, tumor-promoting inflammation	n/d	[18]
**EBP**			
Nicastrin (NCSTN) /#	n/d	n/d	PPI

**DHCR7**			
Integrin-linked protein kinase (ILK) /*	Activating invasion and metastasis, sustaining proliferative signaling	n/d	PPI
E3 ubiquitin-protein ligase listerin (LTN1) / ✦	n/d	n/d	PPI
Tyrosine-protein phosphatase non-receptor type 5 (PTPN5) /**	Activating invasion and metastasis, sustaining proliferative signaling	MAPK	PPI
TRAF2 and NCK-interacting protein kinase (TNIK) /*	n/d	n/d	PPI
Ubiquitin carboxyl-terminal hydrolase CYLD (CYLD) /✦✦	n/d	Toll-like receptor signaling	PPI
cAMP-dependent protein kinase (PRKACA)/*	Activating invasion and metastasis, enabling replicative Immortality, evading growth suppressors, evading immune destruction, inducing angiogenesis, reprogramming energy metabolism, sustaining proliferative signaling, tumor-promoting inflammation	MAPK, Ras, chemokine, Wnt, insulin resistance proteoglycans in cancer	[19]
E3 ubiquitin-protein ligase synoviolin (SYVN1) /✦	n/d	n/d	[18]
E3 ubiquitin-protein ligase (AMFR) /✦	n/d	n/d	[18]
E3 ubiquitin-protein ligase CHIP (STUB1) /✦	n/d	n/d	[18]
E3 ubiquitin-protein ligase HECTD3 (HECTD3) /✦	n/d	n/d	[18]
Probable E3 ubiquitin-protein ligase HERC3 (HERC3) /✦	n/d	n/d	[18]
E3 ubiquitin-protein ligase HUWE1 (HUWE1) /✦	n/d	n/d	[18]
E3 ubiquitin-protein ligase Praja-2 (PJA2) /✦	n/d	n/d	[18]
NEDD4-like E3 ubiquitin-protein ligase WWP2 (WWP2) /✦	n/d	n/d	[18]

(*/**) Phosphorylation / De-phosphorylation; (#) Proteolytic cleavage; (✦/✦✦) Ubiquitylation/De-ubiquitylation; (■) Glycosylation; [1]Cancer Hallmark Genes tool (http://bio-bigdata.hrbmu.edu.cn/CHG/ accessed on 3 March 2021); [2]KEGG pathways (https://www.kegg.jp/kegg/pathway.html accessed on 3 March 2021); [3]PPI - protein-protein interaction databases

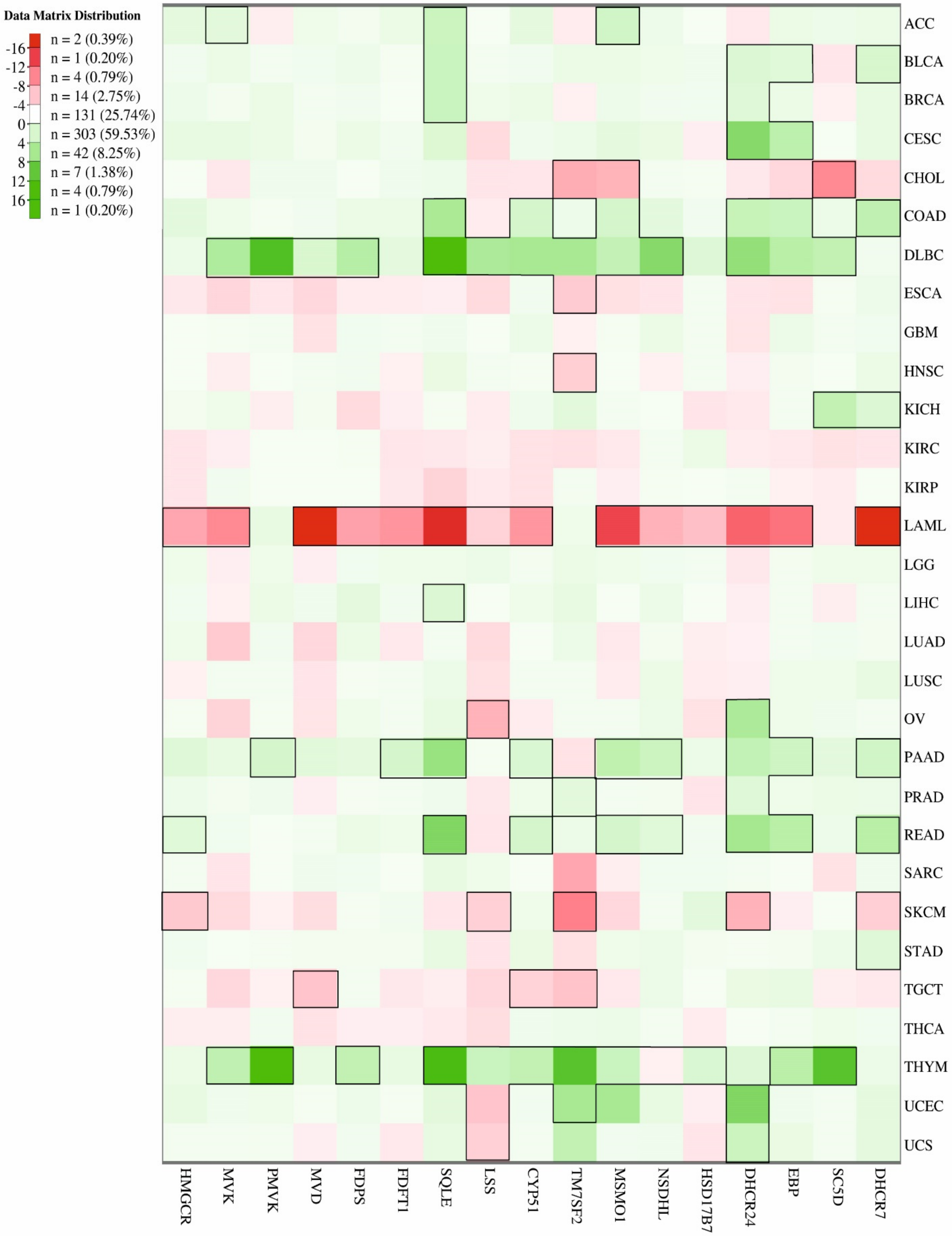

Figure S1. Heat map of expression of 17 genes, encoding cholesterol synthesis enzymes, in tumors with correction for normal tissues. Statistically significant differentially expressed genes (fold change > 3, p-value < 0.01) are highlighted in borders. Down-regulated and up-regulated genes are shown in red and green color, respectively.

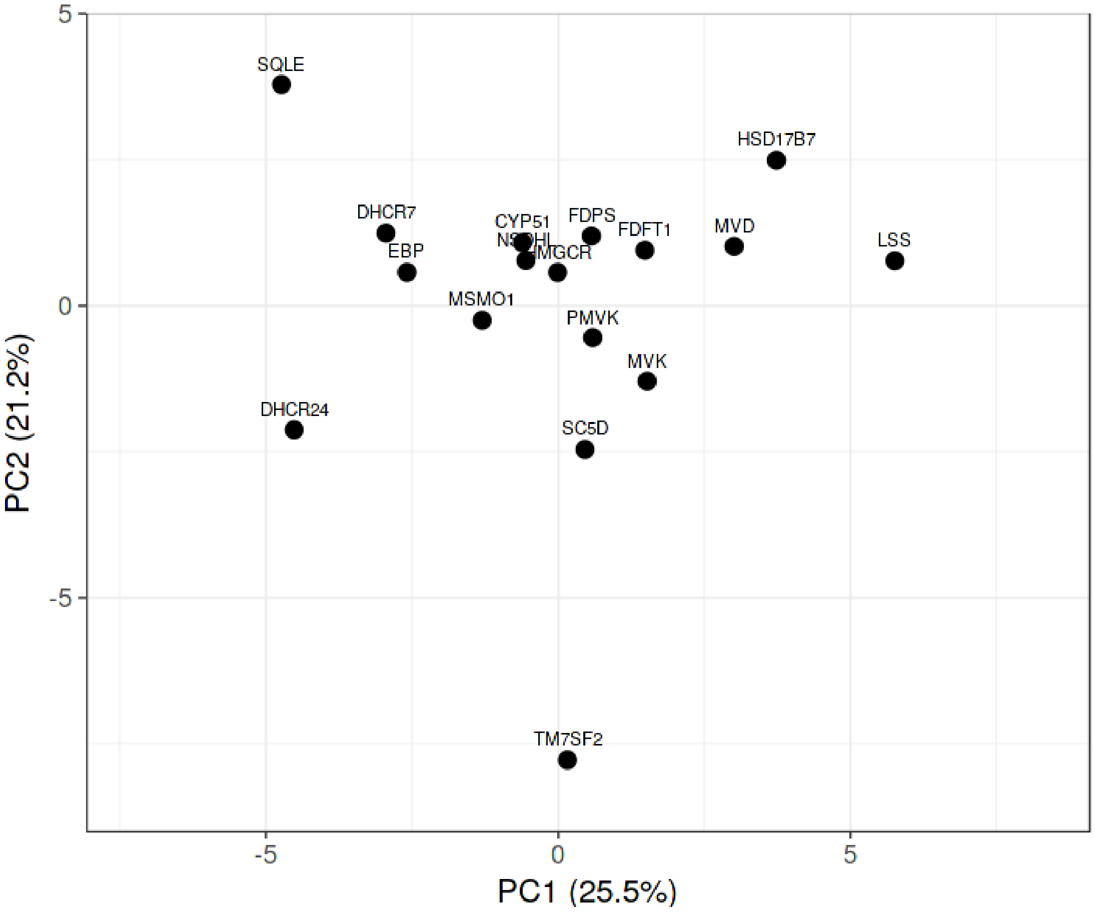

Figure S2. Principle component analysis (PCA) of genes, encoding cholesterol synthesis enzymes, in 30 different tumors.

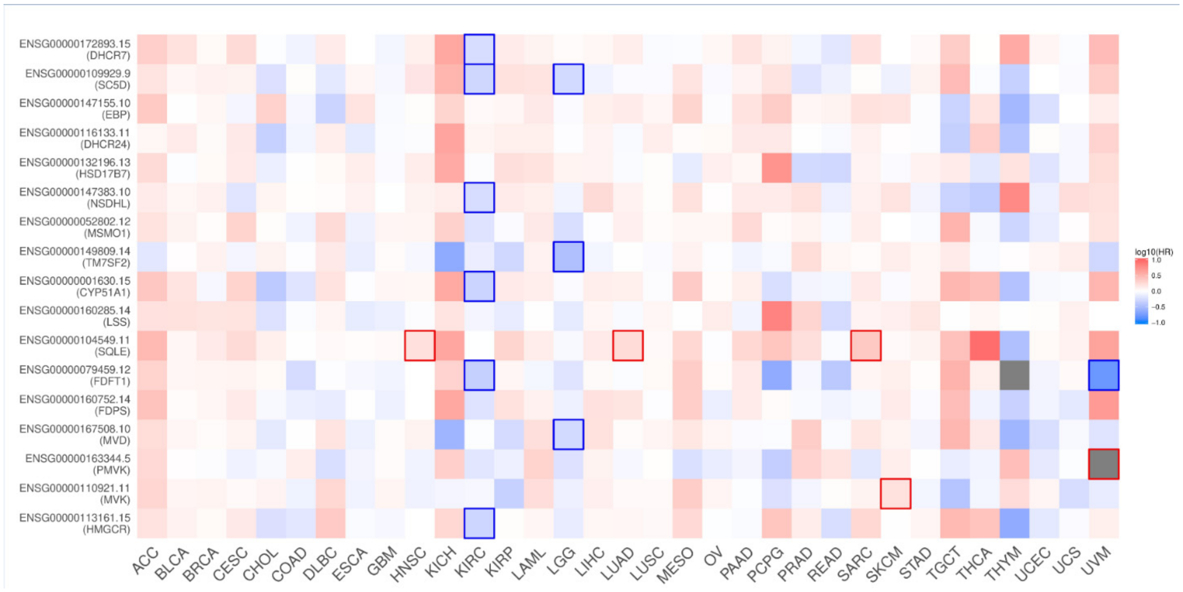

Figure S3. Prognostic value (overall survival) of differentially expressed genes (DEGs), encoding cholesterol synthesis enzymes. Statistically significant gene expression is highlighted with borders.

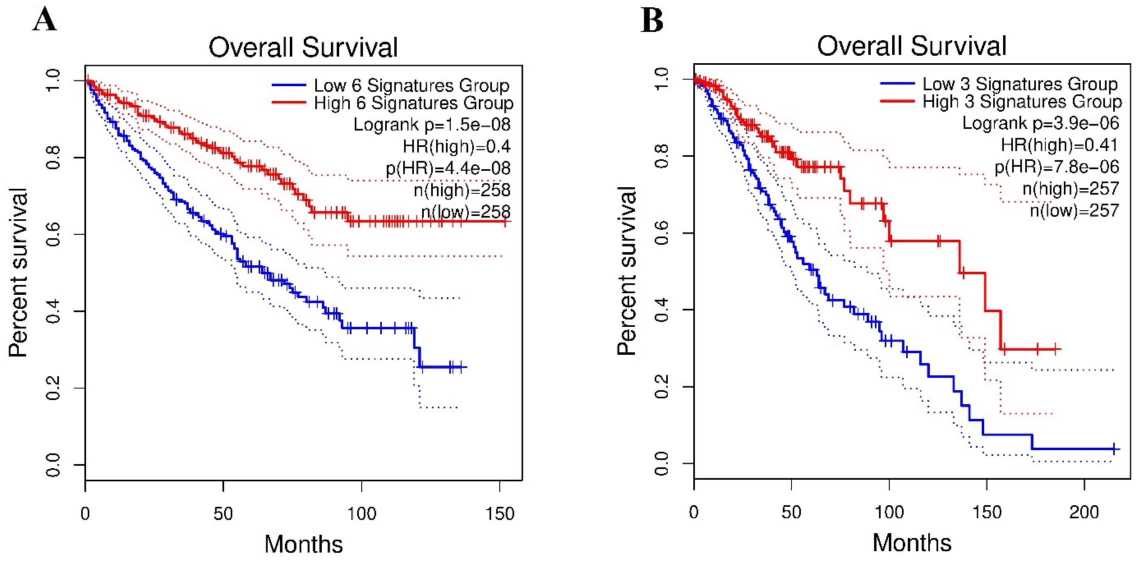

Figure S4. Kaplan-Meier survival plots for the gene signature #1 (**A**) in kidney renal clear cell carcinoma (KIRC) and signature #2 (**B**) in brain lower grade glioma (LGG).

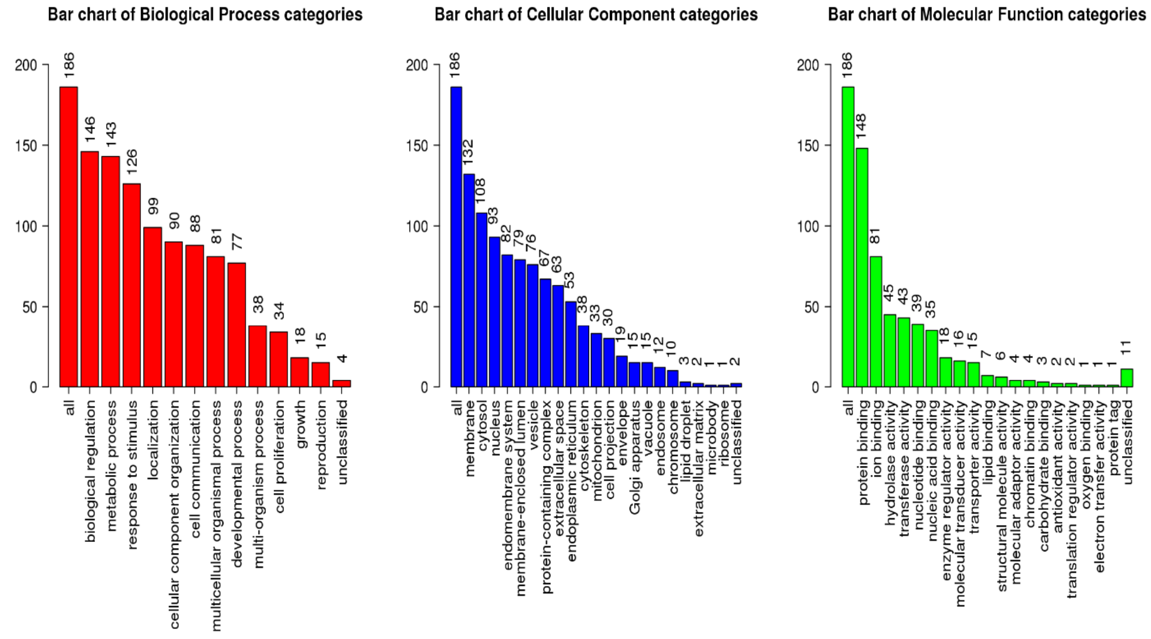

Figure S5. Quantitative distribution of the entire pool of protein partners of the cholesterol synthesis enzymes by cellular components, molecular functions and biological processes according to Gene Onthology terms.

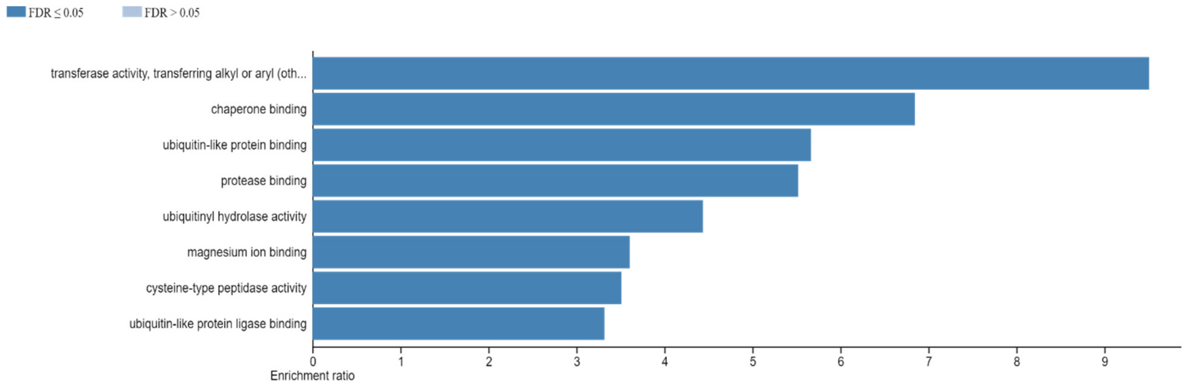

Figure S6. Over-representation analysis of the entire pool of protein partners of the cholesterol synthesis enzymes with molecular function terms.

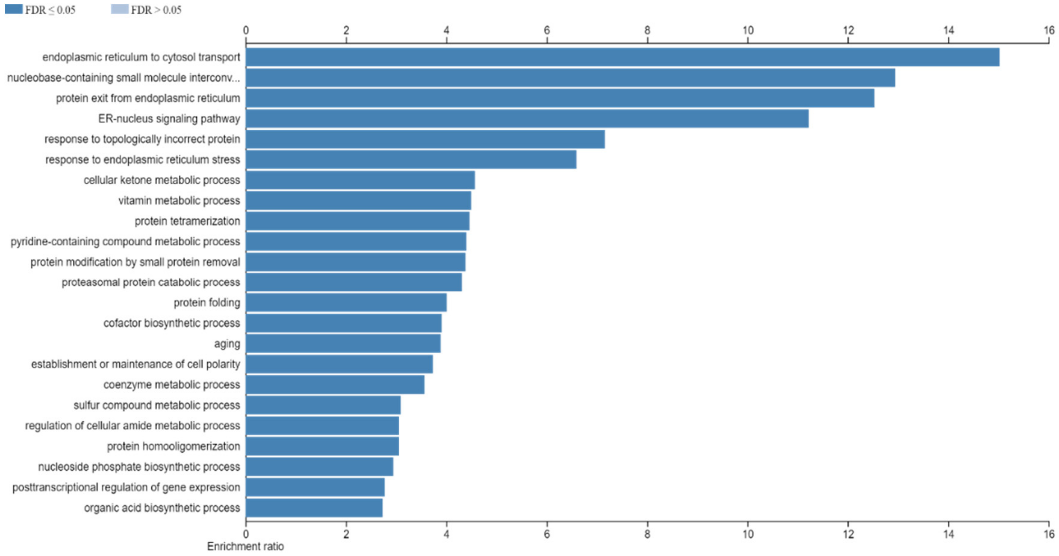

Figure S7. Over-representation analysis of the entire pool of protein partners of the cholesterol synthesis enzymes with biological process terms.

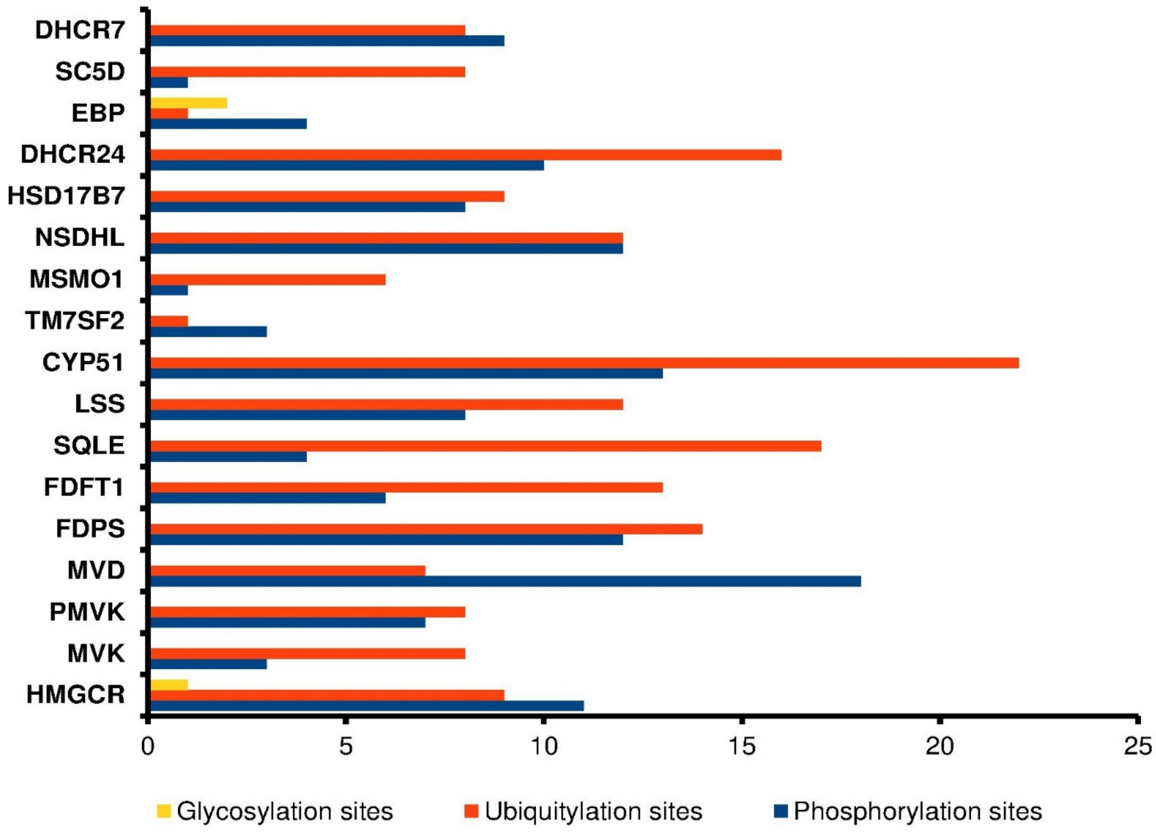

Figure S8. Distribution of mapped phosphorylation and ubiquitylation sites in the cholesterol synthesis enzymes (PhosphoSitePlus database).

References

1. Loh, K.; Tam, S.; Murray-Segal, L.; Huynh, K.; Meikle, P.J.; Scott, J.W.; van Denderen, B.; Chen, Z.; Steel, R.; LeBlond, N.D.; et al. Inhibition of Adenosine Monophosphate-Activated Protein Kinase-3-Hydroxy-3-Methylglutaryl Coenzyme A Reductase Signaling Leads to Hypercholesterolemia and Promotes Hepatic Steatosis and Insulin Resistance. *Hepatol Commun* **2019**, *3*, 84–98, doi:10.1002/hep4.1279.
2. Zhang, X.; Song, Y.; Feng, M.; Zhou, X.; Lu, Y.; Gao, L.; Yu, C.; Jiang, X.; Zhao, J. Thyroid-Stimulating Hormone Decreases HMG-CoA Reductase Phosphorylation via AMP-Activated Protein Kinase in the Liver. *J. Lipid Res.* **2015**, *56*, 963–971, doi:10.1194/jlr.M047654.
3. Wang, Z.; Wang, N.; Liu, P.; Xie, X. AMPK and Cancer. *Exp Suppl* **2016**, *107*, 203–226, doi:10.1007/978-3-319-43589-3_9.
4. Ching, Y.P.; Kobayashi, T.; Tamura, S.; Hardie, D.G. Specificity of Different Isoforms of Protein Phosphatase-2A and Protein Phosphatase-2C Studied Using Site-Directed Mutagenesis of HMG-CoA Reductase. *FEBS Lett.* **1997**, *411*, 265–268, doi:10.1016/s0014-5793(97)00712-6.
5. Wei, H.; Zhang, H.-L.; Xie, J.-Z.; Meng, D.-L.; Wang, X.-C.; Ke, D.; Zeng, J.; Liu, R. Protein Phosphatase 2A as a Drug Target in the Treatment of Cancer and Alzheimer's Disease. *Curr. Med. Sci.* **2020**, *40*, 1–8, doi:10.1007/s11596-020-2140-1.
6. Moriyama, T.; Wada, M.; Urade, R.; Kito, M.; Katunuma, N.; Ogawa, T.; Simoni, R.D. 3-Hydroxy-3-Methylglutaryl Coenzyme A Reductase Is Sterol-Dependently Cleaved by Cathepsin L-Type Cysteine Protease in the Isolated Endoplasmic Reticulum. *Arch. Biochem. Biophys.* **2001**, *386*, 205–212, doi:10.1006/abbi.2000.2209.
7. Jiang, L.-Y.; Jiang, W.; Tian, N.; Xiong, Y.-N.; Liu, J.; Wei, J.; Wu, K.-Y.; Luo, J.; Shi, X.-J.; Song, B.-L. Ring Finger Protein 145 (RNF145) Is a Ubiquitin Ligase for Sterol-Induced Degradation of HMG-CoA Reductase. *J. Biol. Chem.* **2018**, *293*, 4047–4055, doi:10.1074/jbc.RA117.001260.
8. Song, B.-L.; Sever, N.; DeBose-Boyd, R.A. Gp78, a Membrane-Anchored Ubiquitin Ligase, Associates with Insig-1 and Couples Sterol-Regulated Ubiquitination to Degradation of HMG CoA Reductase. *Mol. Cell* **2005**, *19*, 829–840, doi:10.1016/j.molcel.2005.08.009.
9. Lu, X.-Y.; Shi, X.-J.; Hu, A.; Wang, J.-Q.; Ding, Y.; Jiang, W.; Sun, M.; Zhao, X.; Luo, J.; Qi, W.; et al. Feeding Induces Cholesterol Biosynthesis via the MTORC1-USP20-HMGCR Axis. *Nature* **2020**, *588*, 479–484, doi:10.1038/s41586-020-2928-y.

10. Adam, C.; Glück, L.; Ebert, R.; Goebeler, M.; Jakob, F.; Schmidt, M. The MEK5/ERK5 Mitogen-Activated Protein Kinase Cascade Is an Effector Pathway of Bone-Sustaining Bisphosphonates That Regulates Osteogenic Differentiation and Mineralization. *Bone* **2018**, *111*, 49–58, doi:10.1016/j.bone.2018.03.020.
11. Jun, S.Y.; Brown, A.J.; Chua, N.K.; Yoon, J.-Y.; Lee, J.-J.; Yang, J.O.; Jang, I.; Jeon, S.-J.; Choi, T.-I.; Kim, C.-H.; et al. Reduction of Squalene Epoxidase by Cholesterol Accumulation Accelerates Colorectal Cancer Progression and Metastasis. *Gastroenterology* **2021**, *160*, 1194-1207.e28, doi:10.1053/j.gastro.2020.09.009.
12. Loregger, A.; Cook, E.C.L.; Nelson, J.K.; Moeton, M.; Sharpe, L.J.; Engberg, S.; Karimova, M.; Lambert, G.; Brown, A.J.; Zelcer, N. A MARCH6 and IDOL E3 Ubiquitin Ligase Circuit Uncouples Cholesterol Synthesis from Lipoprotein Uptake in Hepatocytes. *Mol. Cell Biol.* **2016**, *36*, 285–294, doi:10.1128/MCB.00890-15.
13. Tan, J.M.E.; van der Stoel, M.M.; van den Berg, M.; van Loon, N.M.; Moeton, M.; Scholl, E.; van der Wel, N.N.; Kovačević, I.; Hordijk, P.L.; Loregger, A.; et al. The MARCH6-SQLE Axis Controls Endothelial Cholesterol Homeostasis and Angiogenic Sprouting. *Cell Rep.* **2020**, *32*, 107944, doi:10.1016/j.celrep.2020.107944.
14. Zelcer, N.; Sharpe, L.J.; Loregger, A.; Kristiana, I.; Cook, E.C.L.; Phan, L.; Stevenson, J.; Brown, A.J. The E3 Ubiquitin Ligase MARCH6 Degrades Squalene Monooxygenase and Affects 3-Hydroxy-3-Methyl-Glutaryl Coenzyme A Reductase and the Cholesterol Synthesis Pathway. *Mol. Cell Biol.* **2014**, *34*, 1262–1270, doi:10.1128/MCB.01140-13.
15. Foresti, O.; Ruggiano, A.; Hannibal-Bach, H.K.; Ejsing, C.S.; Carvalho, P. Sterol Homeostasis Requires Regulated Degradation of Squalene Monooxygenase by the Ubiquitin Ligase Doa10/Teb4. *Elife* **2013**, *2*, e00953, doi:10.7554/eLife.00953.
16. Scott, N.A.; Sharpe, L.J.; Capell-Hattam, I.M.; Gullo, S.J.; Luu, W.; Brown, A.J. The Cholesterol Synthesis Enzyme Lanosterol 14α-Demethylase Is Post-Translationally Regulated by the E3 Ubiquitin Ligase MARCH6. *Biochem. J.* **2020**, *477*, 541–555, doi:10.1042/BCJ20190647.
17. Luu, W.; Zerenturk, E.J.; Kristiana, I.; Bucknall, M.P.; Sharpe, L.J.; Brown, A.J. Signaling Regulates Activity of DHCR24, the Final Enzyme in Cholesterol Synthesis. *J. Lipid Res.* **2014**, *55*, 410–420, doi:10.1194/jlr.M043257.
18. Capell-Hattam, I.M.; Sharpe, L.J.; Qian, L.; Hart-Smith, G.; Prabhu, A.V.; Brown, A.J. Twin Enzymes, Divergent Control: The Cholesterogenic Enzymes DHCR14 and LBR Are Differentially Regulated Transcriptionally and Post-Translationally. *J. Biol. Chem.* **2020**, *295*, 2850–2865, doi:10.1074/jbc.RA119.011323.
19. Prabhu, A.V.; Luu, W.; Sharpe, L.J.; Brown, A.J. Phosphorylation Regulates Activity of 7-Dehydrocholesterol Reductase (DHCR7), a Terminal Enzyme of Cholesterol Synthesis. *J. Steroid Biochem. Mol. Biol.* **2017**, *165*, 363–368, doi:10.1016/j.jsbmb.2016.08.003.
